# Persistence in soil of *Miscanthus* biochar in laboratory and field conditions

**DOI:** 10.1371/journal.pone.0184383

**Published:** 2017-09-05

**Authors:** Daniel P. Rasse, Alice Budai, Adam O’Toole, Xingzhu Ma, Cornelia Rumpel, Samuel Abiven

**Affiliations:** 1 Department of Soil Quality and Climate Change, Norwegian Institute of Bioeconomy Research, Ås, Norway; 2 Department of Environmental Sciences, Norwegian University of Life Sciences, Ås, Norway; 3 Institute of Soil Fertilizer and Environment Resource, Heilongjiang Academy of Agricultural Sciences, Harbin, China; 4 CNRS, IEES, UMR CNRS-INRA-UPMC-UPEC-IRD-ParisAgroTech, Thiverval-Grignon, France; 5 Department of Geography, University of Zurich, Zurich, Switzerland; RMIT University, AUSTRALIA

## Abstract

Evaluating biochars for their persistence in soil under field conditions is an important step towards their implementation for carbon sequestration. Current evaluations might be biased because the vast majority of studies are short-term laboratory incubations of biochars produced in laboratory-scale pyrolyzers. Here our objective was to investigate the stability of a biochar produced with a medium-scale pyrolyzer, first through laboratory characterization and stability tests and then through field experiment. We also aimed at relating properties of this medium-scale biochar to that of a laboratory-made biochar with the same feedstock. Biochars were made of *Miscanthus* biomass for isotopic C-tracing purposes and produced at temperatures between 600 and 700°C. The aromaticity and degree of condensation of aromatic rings of the medium-scale biochar was high, as was its resistance to chemical oxidation. In a 90-day laboratory incubation, cumulative mineralization was 0.1% for the medium-scale biochar *vs*. 45% for the *Miscanthus* feedstock, pointing to the absence of labile C pool in the biochar. These stability results were very close to those obtained for biochar produced at laboratory-scale, suggesting that upscaling from laboratory to medium-scale pyrolyzers had little effect on biochar stability. In the field, the medium-scale biochar applied at up to 25 t C ha^-1^ decomposed at an estimated 0.8% per year. In conclusion, our biochar scored high on stability indices in the laboratory and displayed a mean residence time > 100 years in the field, which is the threshold for permanent removal in C sequestration projects.

## Introduction

Progress towards implementing biochar as a technology for biological carbon capture and storage is being made on several fronts. A recent analysis indicates that biochar is on average a more favorable option than other negative emission technologies in terms of required land surface, water use, soil nutrient budgets, energy requirements and costs [1]. Early fears and uncertainty about the impact of large-scale biochar deployment have been tempered by extensive work to assess possible negative impacts and tradeoffs [2–4] and the creation of industry certification protocols to ensure sustainable production of safe biochar e.g. European Biochar Certificate [5] and biochar standards of the International Biochar Initiative [6]. For C-credit accounting, biochar potentially presents the considerable advantage as compared to other soil carbon sequestration methods of relying on direct C-input accounting rather than expensive soil-based verification schemes [7]. However, C-input accounting is conditional to having an accurate estimator of the mean residence time (MRT) in soil of any given biochar source.

Persistence in soil is a fundamental quality of biochars for serving their role as C sequestration products. This persistence must exceed 100 years to match the definition of permanent removal, as defined by Noble and colleagues [8]. The bulk of plant residue biomass decomposes quickly when applied to soil, with even lignin molecules mineralizing at 90% within one year of residue application to soil [9]. The mean residence time of bulk soil organic matter (SOM) averages 50 years across studies [10]. In other words, biochar must be about 2 orders of magnitude more stable than untreated plant residues and at least twice as stable as bulk SOM to meet a 100-year MRT criteria.

Research on biochar is often carried out using laboratory-produced biochar. Due to limitations of heat transfer and the exothermic nature of pyrolysis, small-scale production offers better control and more sensitive monitoring as compared to larger scale commercial units [11]. The implementation of biochar technology is dependent on the production of biochar through larger scale commercial units. The highest treatment temperature reached during transformation is often different from the target temperature due to the endothermic and exothermic properties of the carbonization process [12], and accurate measurement of temperatures within the reactors are not always possible, especially large-scale ones. This raises the question whether biochar produced in larger reactors is of equivalent quality to that produced in the laboratory using the same feedstock and equivalent temperature.

Up to now, the vast majority of studies aiming at determining the stability of biochar in soils have been laboratory incubations. Reviews of biochar stability in soils have mostly been based on laboratory incubations and on properties of black carbon present in soils exposed to natural fires [13]. In a review of 311 papers, Gurwick and colleagues [14] found only 3 studies estimating biochar stability in the field. Similarly, less than 10% of studies presented in a recent review of biochar effects on soil respiration were based on actual field treatments [15]. Only a subset of these field treatments corresponded to CO_2_ field monitoring for at least one growing season. Recently, only three isotopic field studies were available for estimating biochar decomposition and priming effects in soils, while many more came from laboratory conditions [16]. This exemplifies the need for more field evaluation of biochar, especially as its mineralization might be enhanced in field, where active roots are present [17].

One of the problems with laboratory incubations is the fact that they are usually lasting for a few weeks or months and they are addressing the timeframe of 100 years only by extrapolation of the C mineralization data [18]. Field data are needed to improve upon these extrapolations and to calibrate screening methods for biochar stability [19]. Chemical oxidation is such a screening method, which has been proposed to address long-term biochar stability [20, 21]. Another approach is based on the determination of benzene polycarboxylic acids (BPCA) as biomarkers of condensed aromatic sheets, which have been shown to isolate the most stable faction of biochar, and are therefore a promising proxy for stability [22]. Moreover, elemental composition of biochars may also be a proxy for their degradation behavior [23]. Here we considered these three types of proxies for biochar incubated under both laboratory and field conditions.

The overall objective of the present study was to investigate the stability in soils of biochar produced from *Miscanthus* feedstock. The feedstock was chosen because it is a bioenergy crop in Europe and, being a C_4_-type grass, its distinct isotopic ^13^C signature can be used to trace the fate of its constitutive carbon in temperate soils. Biochars were produced with slow pyrolysis at different scales, using a medium-scale pyrolyzer (BC_MED_) and a laboratory unit (BC_LAB_), and their stabilities were analysed with different laboratory methods and compared to laboratory and field incubation results. The objectives of this study were to: 1) determine if BC_MED_ performed as well as BC_LAB_ in terms of carbonization, condensation, chemical stability indicators and biological stability in laboratory incubation, and 2) estimate the stability of BC_MED_ and its feedstock in a 2-year field experiment.

## Material and methods

### Biochar production and characterization

The *Miscanthus* biochar was produced in Germany in 2010 by Pyreg® Gmbh (www.pyreg.de) in a commercial prototype slow pyrolysis screw reactor operating under a continuous feeding rate of 100–150 kg dry matter per hour and a carbon efficiency of up to 60%. We define this pyrolyzer unit as being of a medium scale and refer to it hereafter as BC_MED_. The estimated highest treatment temperature (HTT) provided by the manufacturer was between 500–750°C. A precise temperature measurement at each phase of pyrolysis is in general difficult to obtain due to heat transfer limitations and was not possible for this machine. In order to avoid combustion risks, the biochar was moistened to approximately 35% moisture content after leaving the pyrolysis reactor. Application rates in this article were all corrected for moisture and are presented on a dry weight basis. Using the same feedstock as BC_MED_, we produced slow-pyrolysis biochars under controlled laboratory conditions and obtained a measured HTT of 682°C. This was performed in a muffle furnace with a heating rate of 2.5°C min^-1^ as described by Budai and colleagues [12]. Hereafter, we will refer to this biochar as BC_LAB_.

The *Miscanthus* biochar was analyzed for elemental and proximate compositions. Proximate analyses for volatile matter content were conducted according to ASTM E 871 and 872 except that covered crucibles were placed at the rear of a furnace and heated for 6 minutes at 950°C, and ash content was determined according to ASTM D 1102. Specific surface area was measured by N adsorption–desorption isotherms at 77 K using a Micromeritics Tri Star 3000 instrument. Before analysis, the samples were dried at 120°C and degassed overnight in a VacPrep 061 Degasser at 0.05 mbar and 393 K. The Brunauer–Emmet–Teller equation was used to calculate the specific surface area [24]. The C and N contents were determined on a Leco CHN 1000 analyzer (Leco Corp., St. Joseph, MI, USA).

Aromaticity and condensation degree of the *Miscanthus* biochars were estimated with the method of BPCA, following Wiedemeier and colleagues [25]. BPCAs are molecular markers that originate from larger aromatic structures that compose charred biomass. The quantity and composition of the BPCA molecular markers are used to deduce information about the molecular structure of biochar. Here we used total BPCA amount in relation to organic carbon (g kg^-1^) as an indicator of aromaticity and the ratio of B6CA per total BPCA as an indicator of condensation, as suggested by Wiedemeier and colleagues [26]. The BPCA method was carried out by digesting each ball-milled sample in 10, 15, and 20 mg aliquots for 8 hours at 170°C in quartz tubes using 2 mL of 65% nitric acid solution. The digestate was filtered through ash-free cellulose paper and a cation exchange resin, then finally freeze-dried and re-dissolved in methanol/water (1:1, v:v) before passing through a conditioned solid phase extraction column (Supelco, USA). After drying and re-dissolving in ultrapure water, the final sample was analyzed using an Agilent 1290 Infinity HPLC system (Santa Clara, USA) according to Wiedemeier and colleagues [25].

Resistance of biochar to oxidation was tested by the acid dichromate method as described by Naisse and colleagues [20], where the total length of time for oxidation is chosen according to the time required to oxidize all of a reference material, i.e. the feedstock in this case. The method of applying fresh potassium dichromate solution and allowing for variable reaction time was applied by Rumpel and colleagues [27] and applied also at room temperature by Kuo and colleagues [28]. Here, samples of 0.3 g each were oxidized in 5 mL of 0.1 M K_2_Cr_2_O_7_ / 2M H_2_SO_4_ for 1.5 or 2 hours under sonication at 70°C. Samples were recovered by centrifugation and the removal of supernatant, after which oxidation was repeated with new potassium dichromate acid solution. Oxidation was repeated until the *Miscanthus* feedstock was consumed. Total oxidation time was 15.5 hours for all samples. Remaining samples were washed three times with 5 ml distilled water and dried at 60°C for two days. Sample remnants were ground using a mortar and pestle before C and N analysis.

### Incubation

Incubation was carried out using a sandy loam Inceptisol collected from an agricultural field in Rygge county, Norway (59°23′15″ N; 10°46′26″ E) [29]. Soil consisted of 83% sand, 11% silt, and 6% clay (Eurofins AS, Norway), had a pH of 6.8 as measured at a 1:1 soil to water ratio, TOC content of 12 g kg^-1^ (dw), and a C/N ratio of 12. This soil does not come from our biochar field experiment, but it is a standard soil we used for laboratory incubation of our biochar series [30]. Because of this difference in soil type, our laboratory incubations of BC_MED_ are not directly comparable to mineralization under field conditions, but rather provide a realistic use of laboratory incubation as a proxy for field stability, where incubation would most likely not be conducted in each soil type where field application is considered. The air-dried soil was passed through a 2 mm sieve, brought to 19.8% (g g^-1^) moisture content and pre-incubated at 20°C for 20 days. Feedstock and biochars were added to 20 g equivalent dry soil at rates of 0.025, 0.12, 0.58% for feedstock and 0.23, 1.14, 5.46% for biochars. For biochars, these rates mimicked application rates of about 6, 30 and 150 t BC ha^-1^ within a 0.20 m soil layer of bulk density 1300 kg m^-3^ for the untreated soil. In order to adjust for the faster mineralization of the feedstock, application rate was 10% that of biochar. All mineralization rates were computed based on precise amount added to each vial. The first two rates are close to those used in our field experiments, while the high rate provides an end member for testing potential dose dependent effects on mineralization. Incubation was carried out in 120 mL incubation vials equipped with butyl rubber septa. Determination of the accumulated CO_2_ concentration and ^13^CO_2_ signature was conducted every 7 to 11 days according to the batch-flush method as described in Budai and colleagues [30]. In short, sampling was conducted by flushing the vial headspace with 800 mL CO_2_-free air with outflow gasses collected in 1L gasbags. The concentration and ^13^C isotopic composition in the gasbag was then measured using a cavity ring-down spectrometer (G1101-i, Picarro, INC., Sunnyvale, CA, USA), which had been factory upgraded to reduce transient concentration response, water vapor interference and CH_4_ interference according to Moni and colleagues [31]. In addition, a Nafion filter with desiccator was installed on-line to further reduce possible interaction with water vapor [30].

### Field trial

A field trial was set up in September 2010 in Ås, Norway (59° 39' 51" N 10° 45' 40" E) in a randomized block design with 4 treatments x 4 blocks. Plots were 8 x 4 m and buffer areas between blocks were 6 m wide. The 4 treatments consisted of: (1) BC_MED_ biochar at 8 t C ha^-1^ (BC8), (2) BC_MED_ biochar at 25 t C ha^-1^ (BC25), (3) *Miscanthus* straw (non-pyrolyzed) at 8 t C ha^-1^ (MS8), and (4) control (neither biochar nor non-pyrolyzed *Miscanthus*). Application rates were computed per unit C so that equal quantities of C were added in BC8 and MS8 treatments. Biochar and *Miscanthus* straw were hand spread and raked out on the surface of the plots in September 2010, and immediately incorporated into the soil by inverse ploughing. Inverse ploughing to a depth of 23 cm resulted in the biochar and straw being distributed in concentrated diagonal seams in the Ap horizon in 2011. Ploughing and harrowing after harvest in 2011 and 2012 resulted in a more even distribution throughout the Ap horizon in the following years. Oats were sown in 2011 and barley in 2012. Fertilization was applied with seeding using Yaramila ™ NPK 22-3-10 at 550 kg fertilizer ha^-1^. The fields were not treated with fungicide, herbicide or pesticide. Hand weeding was done where weeds appeared within the closed chamber collars.

Annual precipitation in 2011 was 973 mm (63% in May-Sept) and 800 mm in 2012 (47% in May-Sept). Annual average temperature was 6.7°C in 2011 (14 ± 2.6°C, May-Sept), and 5.9°C in 2012 (13.1 ± 2.3°C, May-Sept). These meteorological measurements were taken from a research weather station located on the University of Life Sciences, Ås Campus, 1.3 km from the field site. The soil of the field plots is a clay loam Epistagnic Albeluvisol (WRB classification). The clay content is 27%, silt 43% and sand 31%. pH is 6.39 (±0.18, n = 9), TOC 2.64%, total N 0.23%, and total P 0.29%.

### Field CO_2_ monitoring

The CO_2_ fluxes were measured during the growing seasons of 2011 and 2012, and isotopic ^13^C composition in 2012 only. Fourteen CO_2_ flux measurements were undertaken from 23/05/2011 to 01/09/2011 and 11 measurements from 22/05/2012 to 04/10/2012. Measurements were conducted between 10:00 and 15:00. Thirty-two chamber collars (2 collars/plot x 16 plots) measuring 0.32m L x 0.12m W x 0.06 m H were inserted 0.05m into the soil between crop rows, leaving a water filled gutter (0.1m W x 0.1 m H) exposed at the soil surface to serve as a gas sealant for the chamber. The inter-row chambers capture the soil respiration, including root activities, but exclude the respiratory component of plant shoots, thereby increasing the signal to noise ratio of the isotopic measurements. Thirty-two rectangular aluminum closed chambers (0.30m L x 0.1m W x 0.2m H) were placed on the chamber collars immediately before measurement. There were no pressure valve tubes used on the chambers. The CO_2_ flux was measured for 2 min periods with an infrared gas analyzer (IRGA) EGM-4 (PP Systems, Hitchin, UK) which cycled gases via entry and exit valves from the chamber to calculate changes in CO_2_ concentration and the flux.

The δ^13^C signature of the soil CO_2_ efflux was measured 6 times in 2012. Samples were taken in partially inflated 1-L gas bags [31]. Because the air inside the chamber was a mixture of atmospheric air with increasing concentration of soil-emitted CO_2_, keeling plots were necessary to estimate the true δ^13^C value of the soil CO_2_. The keeling plot method [32] is used to differentiate δ^13^C SOM from atmospheric δ^13^C where the linear regression plot intercept represents the δ^13^C SOM. The Keeling plot method is based on a linear relationship between δ^13^C values and the inverse of CO_2_ concentrations, it is therefore not time dependent, making it a robust method even if release of soil air in the chamber might have been slightly accelerated at sampling. Preliminary tests indicated that 3-point keeling plots with sampling at 3, 8 and 1440 minutes were linear and suitable for covering a wide range of concentration necessary for proper estimates. In our analyses, any keeling plot that did not reach a significant correlation coefficient at *P*<0.1 (r ≥ 0.988) was excluded. Gas samples were analyzed for δ^13^C using a cavity ring down spectrometer (G1121-i, Picarro INC., Sunnyvale, CA, USA). Solid sample δ^13^C analysis was carried out on the *Miscanthus* straw, biochar, and the C3 field soil by combusting 1–2 mg samples (3 replicates) in a combustion module connected to a cavity ring-down spectrometer (G2121-i, Picarro, INC., Sunnyvale, CA, USA). The spectrometer was controlled for drift in δ^13^C signal by including known δ^13^C standards, in this case sucrose with -11.6 ‰ and tyrosine at -23.2 ‰, within the analysis runs.

### Statistics

For the laboratory incubations, in order to determine if SOM decomposition was significantly modified by different types and quantities of biochar and feedstock amendment as compared to a non-amended control soil, we applied one-way ANOVA with the Dunnett's method for multiple comparisons vs. a control group, as implemented in SigmaPlot 12.5. Multiple comparisons effects were conducted after verifying that both normality and normal variance conditions were satisfied. Fitting of incubation data to a first-order kinetics decay model was conducted with SigmaPlot 12.5. For field data, statistical analyses of the total soil respiration and mineralization of feedstock and biochar were conducted by 2-way ANOVA, considering treatment and block effects, using the Holm-Sidak method for multiple comparisons when a main effect was detected and both normality and normal variance conditions were satisfied.

## Results

### Laboratory analysis

The BC_MED_ produced at a reported temperature between 500–750°C appeared pyrolyzed to an equivalent extent as compared to our reference 682°C laboratory-scale slow pyrolysis biochar (BC_LAB_). Volatile matter content was 7.4 and 6.4% for BC_MED_ and BC_LAB_, respectively (Table 1). Carbon content was 80 and 76% for BC_MED_ and BC_LAB_, respectively. The H/C atomic ratio was 0.18 and 0.24 for BC_MED_ and BC_LAB_, respectively.

**Table 1:** Properties of the *Miscanthus* feedstock (MS) and derived slow-pyrolysis biochars from medium-scale pyrolyzer (BC_MED_) and a laboratory unit (BC_LAB_).

Property	Units	MS	BC_MED_	BC_LAB_
Volatile Matter	%	78.0	7.4	6.4
Fixed Carbon	%	13.5	81.1	77.7
Ash	%	8.5	11.5	15.9
C	%	47.9	80.0	75.6
H	%	6.1	1.2	1.5
N	%	0.19	0.6	0.6
O	%	51.0	6.6	5.0
H/C (atomic)	-	1.51	0.18	0.24
O/C (atomic)	-	0.80	0.06	0.05
C recalcitrant to K_2_Cr_2_O_7_	%	-	75.4	74.2

Selective oxidations were conducted until all feedstock C was mineralized by the action of the potassium dichromate. At that time, the fraction of non-oxidized C was 75 and 74% for BC_MED_ and BC_LAB_, respectively (Table 1). Total benzene poly-carboxylic acid content, i.e. the sum of B3CA, B4CA, B5CA and B6CA, was 179 and 176 g BPCA-C per kg biochar C for BC_MED_ and BC_LAB_, respectively (Table 2), indicating high content of aromatic moieties in both biochars. The feedstock contained no B6CA, which is a molecular marker of condensed polyaromatic sheets. By contrast, BC_MED_ contained 136 g B6CA-C per kg biochar C, which was 48% more than BC_LAB_. The ratio of B6CA/BPCA was 0.76 for BC_MED_ and 0.53 for BC_LAB_, respectively. Both B6CA as percent of charcoal C [33] and the ratio B6CA/BPCA [26] have been suggested as predictors of aromatic condensation, indicating that BC_MED_ was more condensed than BC_LAB_.

**Table 2:** Content of BPCA biomarkers in biochars from medium-scale pyrolyzer (BC_MED_) and laboratory unit (BC_LAB_).

	BC_MED_		BC_LAB_	
Total BPCA-C (g kg^-1^ C)	179.1	(±0.7)	175.7	(±1.5)
B6CA (%)	76.0	(±0.7)	52.6	(±0.5)
B5CA (%)	14.0	(±0.1)	27.9	(±0.2)
B4CA (%)	10.0	(±0.5)	18.5	(±0.4)
B3CA (%)	0.0	(±0.3)	1.0	(±0.3)

### Laboratory incubations

**Fig 1:**
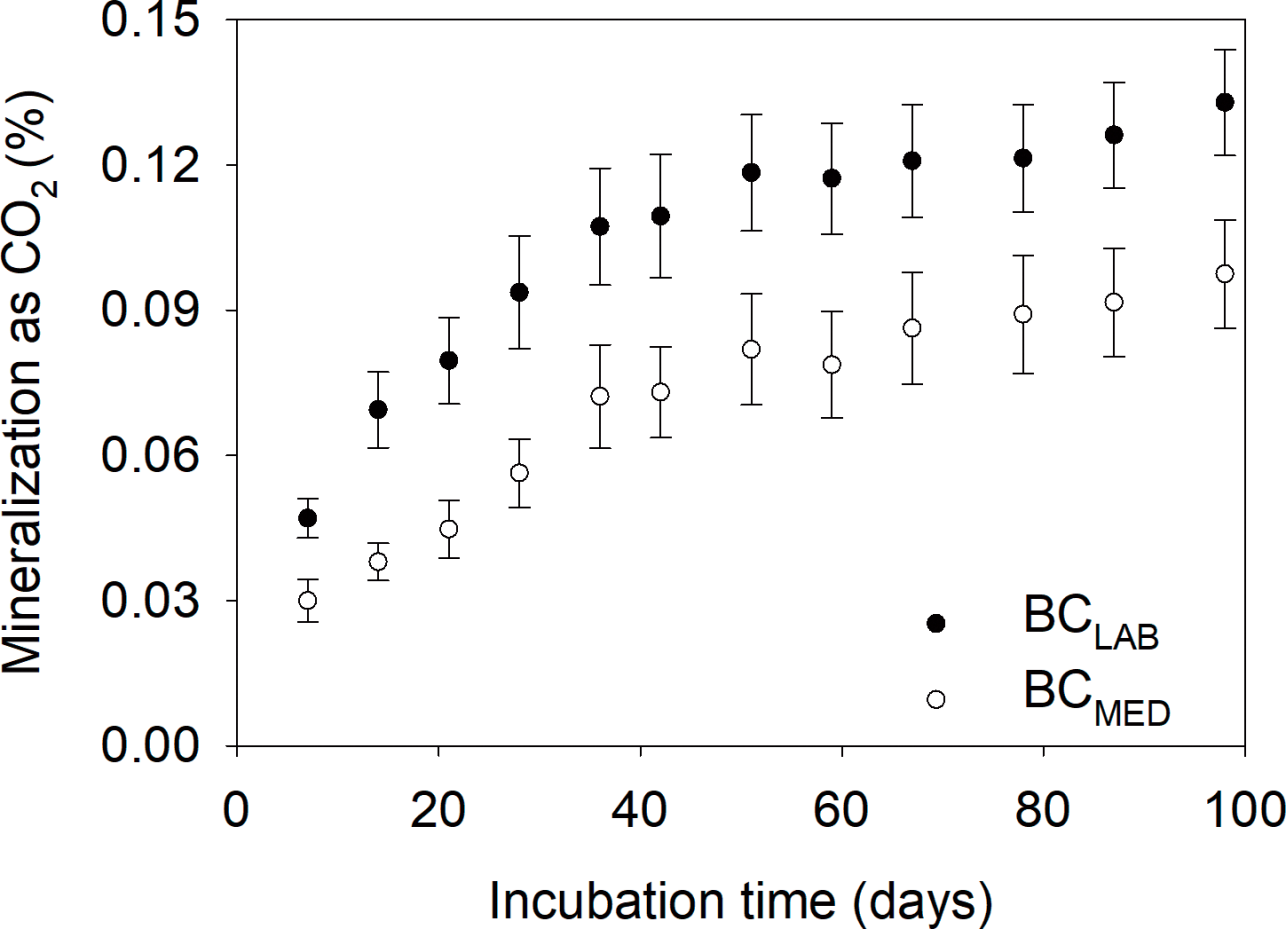
Cumulative mineralization in soil of biochars from medium-scale pyrolyzer (BC_MED_, open symbols) and a laboratory unit (BC_LAB_, filled symbols). Standard errors reported for *n* = 9 (3 replicates for 3 doses). Values are in %, i.e. 100 × mineralized fraction.

In a 90-day incubation, cumulative mineralization of feedstock, SOM and biochar approximated 45%, 1.4% and 0.12% of initial C, respectively (Table 3). This indicates that both biochar types, i.e. BC_MED_ and BC_LAB_, were >300 times more stable than the *Miscanthus* feedstock and >10 times more stable than SOM within the 90-day incubation. Because we were interested in determining if the two biochar types behaved differently, we conducted a 2-way ANOVA biochar × dose for BC_MED_ and BC_LAB_ only (S1 Table). This analysis showed that cumulative mineralization after 90 days was significantly lower for BC_MED_ than for BC_LAB_ (*P = 0*.*03*, S1 Table), while there was neither significant dose effect nor significant dose × biochar interactions. This result suggests that biochar decomposed in a similar fashion whether applied at application rates of 0.23, 1.14 or 5.46% by weight. Feedstock mineralization was also consistent across application rates of 0.03, 0.12 and 0.58% (Table 3). As there was no significant dose effect, we investigated the mineralization kinetics of BC_MED_ vs. BC_LAB_ averaged across doses (Fig 1). This approach shows that the shape of the mineralization curves of BC_MED_ and BC_LAB_ were similar, although total mineralization was significantly lower for BC_MED_ than for BC_LAB_ as mentioned above. Mineralization curves of our two biochars were rather quickly leveling off (Fig 1). In fact, modelling with one-pool first-order kinetics decay, predicted reactive C pool in the BC_MED_ biochar to be 0.10%, with the remaining fraction of about 99.9% being totally inert (S1 Fig). Using a two-pool model yielded similar results. Forcing the one-pool first-order kinetics model to reach 100% mineralization yielded a decay rate of 1.25 10^−5^ d^-1^ or MRT of about 220 years, but the fit to the data was poor (S1 Fig). Therefore, our laboratory incubation simply indicates that BC_MED_ is highly stable and extrapolating a precise MRT remains uncertain.

**Table 3:** Mineralization of C sources at the end of a 90-day incubation of feedstock (MS), BC_MED_ and BC_LAB_ in soils.

C substrate	Dose	Mineralization	
	%	%	
SOM	-	1.38	(±0.02)
MS	0.03	43.1	(±2.6)
	0.12	44.3	(±1.6)
	0.58	46.4	(±0.3)
BC_MED_	0.23	0.13	(±0.04)
	1.14	0.07	(±0.01)
	5.46	0.09	(±0.01)
BC_LAB_	0.23	0.14	(±0.06)
	1.14	0.14	(±0.02)
	5.46	0.12	(±0.00)

Data are percent losses from initial C input, standard deviations for *n* = 3 provided.

Cumulative mineralization of the indigenous SOM was significantly higher in several feedstock and biochar treatments than in the control soil, which averaged 1.38% at the end of the incubation period (Table 4). Largest difference was observed for *Miscanthus* feedstock applied at the highest gravimetric dose with additional loss of 1.08% SOM as compared to the control soil (Table 4). The BC_MED_ applied at ten times this rate, i.e. 5.5%, resulted in a 1% priming of SOM, i.e. an increase in mineralization from 1.4% to 2.4% at the end of the 90-day incubation. All application rates at 5.5% and 1.1% for BC_MED_ and BC_LAB_ biochars and 0.58 and 0.12% for *Miscanthus* feedstock produced a significant increase in SOM mineralization rate as compared to the control. By contrast, no significant difference as compared to control was observed for the lowest amendment rates of 0.23% for BC_MED_ and BC_LAB_ biochars and 0.03% for *Miscanthus* feedstock. For the middle and high amendment doses, which produced significant increases in SOM decomposition, we computed the priming effect and tested for differences among both doses and treatments (Table 5). Both amendment type and dose had significant effects on the cumulative priming rates. The higher amendment dose consistently produced higher priming effects, both for feedstock and biochars (Table 5). Because of a significant amendment × dose interaction (P < 0.01, S2 Table), amendment effects were analyzed within dose. At the middle dose, MS and BC_MED_ induced a similar priming effect, which was significantly higher than that of BC_LAB_. At the higher dose, priming effects were in the order MS > BC_MED_ > BC_LAB_.

**Table 4:** Multiple comparison test for difference of means in SOM mineralization (from indigenous C3 source) in feedstock, and biochar amended vials vs. the non-amendment control.

Comparison	Diff of Means
	(%)
MS @ 0.58% *vs*. soil	1.08***
MS @ 0.12% *vs*. soil	0.24***
MS @ 0.03% *vs*. soil	0.06^ns^
BC_MED_ @ 5.5% *vs*. Soil	1.00***
BC_MED_ @ 1.1% *vs*. Soil	0.26***
BC_MED_ @ 0.2% *vs*. Soil	0.06^ns^
BC_LAB_ @ 5.5% *vs*. soil	0.45***
BC_LAB_ @ 1.1% *vs*. soil	0.12**
BC_LAB_ @ 0.2% *vs*. soil	0.06^ns^

Treatments are *Miscanthus* feedstock (MS) and biochars from medium-scale pyrolyzer (BC_MED_) and laboratory unit (BC_LAB_) (*n* = 3 for all treatments).

** and *** indicate significant differences at *P* < 0.01 and *P* < 0.001, respectively.

ns = non significant.

**Table 5:** Priming effect by *Miscanthus* feedstock (MS) and biochars from medium-scale pyrolyzer (BC_MED_) and laboratory unit (BC_LAB_).

	Priming effect (%)	
Amendment	Middle dose	High dose
MS	17.1^a,A^ (±0.9)	78.5^a,B^ (±2.8)
BC_MED_	19.0^a,A^ (±1.6)	72.4^b,B^ (±1.2)
BC_LAB_	8.6^b,A^ (±0.9)	32.5^c,B^ (±0.7)

Standard errors for *n* = 3 between brackets. Within amendment, different capital letters indicate significant dose effect. Within dose, different small letters indicate significant amendment effect. Significances at *P* < 0.05 for means.

### Field experiment

**Fig 2:**
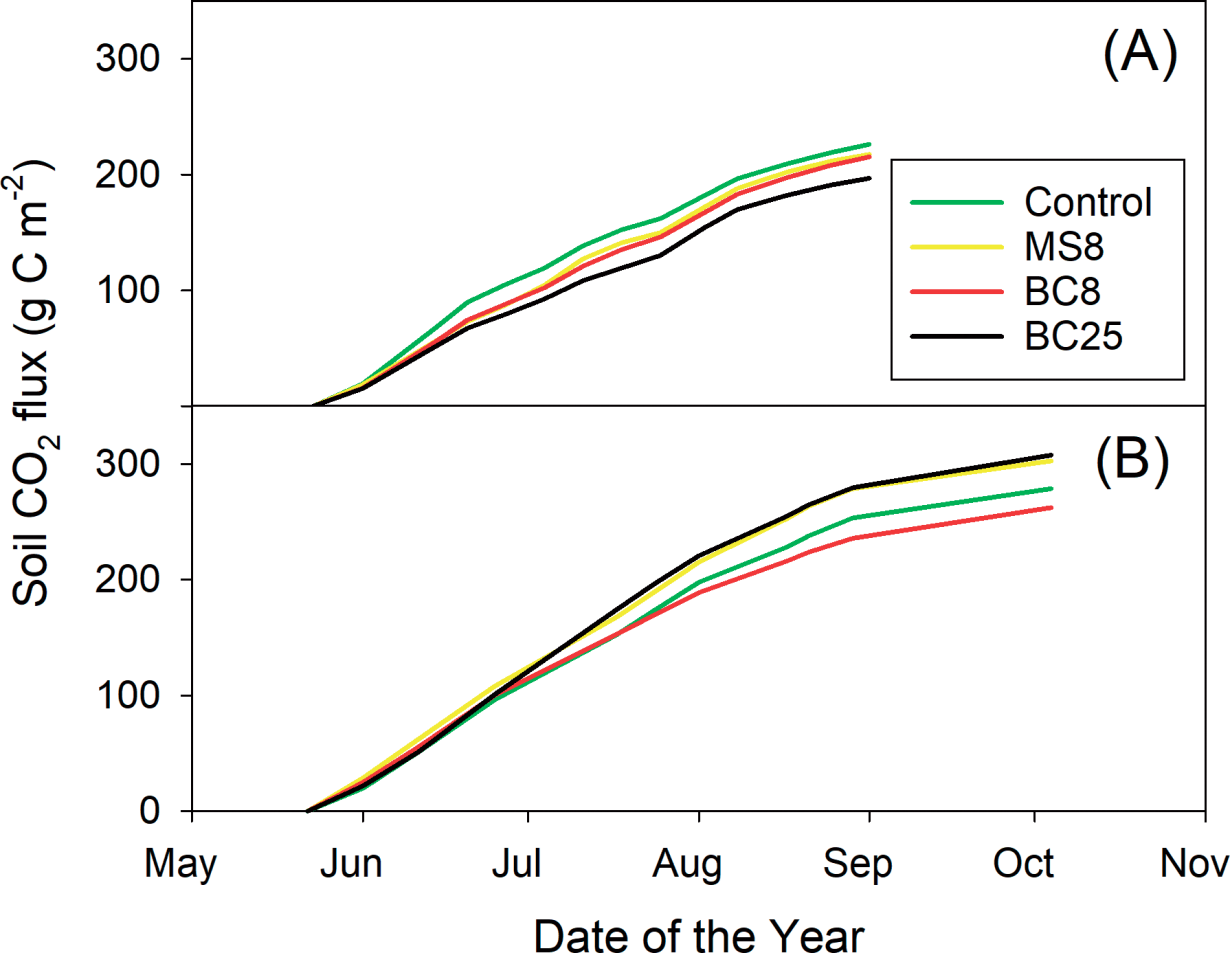
**Cumulative soil respiration measured 14 dates in 2011 (a) and 11 dates in 2012 (b).** Treatments are control (C), non-pyrolyzed Miscanthus feedstock at 8 t C ha^-1^ (MS8), biochar 8 t C ha^-1^ (BC8), and biochar 25 t C ha^-1^ (BC25). Data are averages of n = 4.

Cumulated soil CO_2_ fluxes over the course of the growing season were not significantly affected by treatment in 2011 or 2012, with non-significant highest value in the control treatment in 2011, and in BC25 treatment in 2012 (Table 6, Fig 2). Crop yields were not significantly modified by biochar treatments in either 2011 or 2012 (S3 Table), suggesting that autotrophic respiration terms were fairly similar. Across treatments, cumulated CO_2_ fluxes averaged 214 g C m^-2^ from May 23^rd^ to September 1^st^ in 2011, and 288 g C m^-2^ from May 22^nd^ to October 4^th^ in 2012 (Fig 2).

**Table 6:** Proportion of mineralized C_4_ C source (*Miscanthus* feedstock or biochar) for the 2012 growing season based on the cumulated CO_2_ flux (9 dates) and average δ^13^C values (6 dates).

Treat	Soil CO_2_ flux	δ^13^C	C_4_ CO_2_ in flux	C_4_ CO_2_ total	Mineralized C
	g C m^-2^	‰	%	g C m^-2^	%
C	279 (±29)	-28.2^b^ (±0.2)			
BC8	262 (±31)	-27.8^b^ (±0.1)	2.5^b^ (±0.7)	6.0^b^ (±1.3)	0.75^b^ (±0.16)
BC25	308 (±25)	-27.8^b^ (±0.2)	2.5^b^ (±1.0)	8.0^b^ (±3.7)	0.32^b^ (±0.15)
MS8	303 (±45)	-24.7^a^ (±0.3)	21.9^a^ (±1.8)	66.8^a^ (±12.6)	8.35^a^ (±1.58)

Treatments are control (C), 8 t biochar-C ha^-1^ (BC8), 25 t biochar-C ha^-1^ (BC25) and 8 t *Miscanthus*-C ha^-1^ (MS8). Averages with different superscript letters are significantly different at *P* < 0.05 according to the Holm-Sidak method (*n* = 4 replicated blocks).

For most sampling periods, we obtained highly linear keeling plots for estimating δ^13^C values of the soil CO_2_ (e.g. S2 Fig). The average δ^13^C of soil CO_2_ efflux in the plots amended with *Miscanthus* feedstock was significantly higher than in the control and biochar plots (Table 6). Neither BC8 nor BC25 displayed δ^13^C values of soil CO_2_ significantly different from that of the control, although these values were consistently higher in biochar plots (Table 6). As δ^13^C of BC25 and BC8 were not significantly different from one another, we averaged them per block and compared them to the control. This analysis indicated that the increase in δ^13^C of soil CO_2_ in the biochar plots as compared to the control was not significant at *P* < 0.05 but was so at *P*<0.1 (*P* = 0.06, S4 Table).

**Fig 3:**
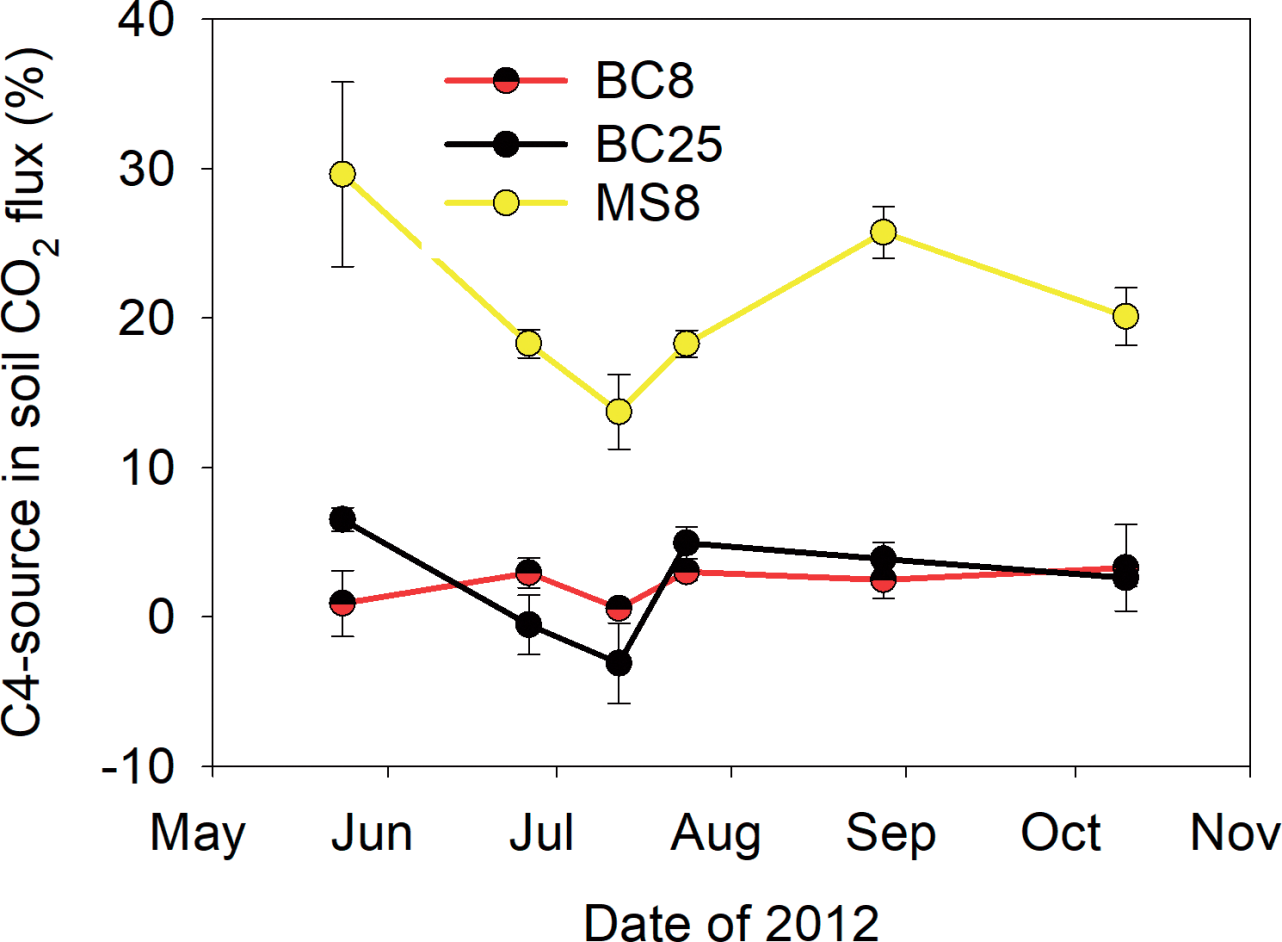
Proportion of the soil CO_2_ efflux coming from the mineralization of 8 t biochar-C ha^-1^ (BC8), 25 t biochar-C ha^-1^ (BC25) and *Miscanthus* straw at 8 t C ha^-1^ (MS8) (standard errors for *n* = 4).

In 2012, the proportion of the soil CO_2_ efflux coming from the *Miscanthus* sources ranged between 15 and 29% for straw, and between 0 and 8% for the biochar (Fig 3). This low contribution of biochar sources appear to mask potential differences between the two dose treatments, with the two curves crossing each other (Fig 3). Although proportions of *Miscanthus*-derived CO_2_ varied during the growing season, no clear seasonal trend was observed (Fig 3), suggesting that a season-average value for the δ^13^C was justified. Because 2012 was the only year with isotopic measurements, we estimated the proportions of *Miscanthus*-derived CO_2_ for the 2012 growing season only (Table 6). Combining measured CO_2_ fluxes and proportions of *Miscanthus*-derived CO_2_, we estimated that our MS8 treatment applied at 800 g C m^-2^ lost 67 g C m^-2^ to the atmosphere during the 2012 growing season, while biochar treatments lost between 6–8 g C m^-2^ during the same period (Table 6). These values correspond to a mineralization of the MS8, BC8 and BC25 by 8.3, 0.8 and 0.3%, respectively. The average mineralization value for *Miscanthus* biochar was therefore 0.5% from May 22 to October 4 in 2012. This value translates into an annual mineralization rate of 0.8%, assuming a Q10 of 2 applied to soil temperature values measured at a depth of 2 cm at the Ås field station.

## Discussion

### Carbonization degree

Laboratory analyses pointed towards equivalent degrees of stability and aromaticity for the medium-scale and the laboratory biochars. The H/C atomic ratio of BC_MED_ was slightly lower than that of BC_LAB_, i.e. 0.18 vs 0.24 (Table 1). Similar to our results, Keiluweit and colleagues [34] reported H/C atomic ratio of 0.2 for grass biochar produced at 700°C, but did not test higher HTT. However, 0.2 is not the lowest limit for biochar produced with *Miscanthus*, as Budai and colleagues [12] report H/C atomic ratio of 0.1 for biochar produced in the laboratory at 800°C. Therefore, the H/C atomic ratio suggests that BC_MED_ reached a carbonization degree comparable to that of BC_LAB_, i.e. a slow-pyrolysis biochar produced in the laboratory at 682°C.

Our chemical oxidation values were close to those reported for a wheat-derived gasification char, which was resistant at 72% to chemical oxidation by potassium dichromate [20]. This latter study used a methodology similar to ours, only with a slightly shorter reaction time, i.e. 12 vs 15.5 h. In general, oxidation methods reported in the literature follow variable protocols, making it difficult to compare results among individual studies. Oxidation utilizing hydrogen peroxide and thermogravimetric analysis have also been used to estimate biochar stability [35]. Our chemical oxidation data suggest that BC_LAB_ and BC_MED_ were equally carbonized.

The BPCA analyses suggest that BC_MED_ produced at a reported temperature between 500–750°C reached a higher condensation degree than our reference 682°C BC_LAB_. Another *Miscanthus* biochar produced by Pyreg was analyzed by Wiedner and colleagues [36] using the BPCA method. Similar to our findings, they found high levels of B6CA, i.e. 85% B6CA, 10% B5CA, 5% B4CA, 0% B3CA. The degree of condensation of this biochar was reported to be higher than all other materials tested [36]. The total BPCA content of our BC_LAB_ and BC_MED_ are similar to those obtained for grass biochars prepared at 700–900°C [33, 37]. Our results suggest that the medium-scale pyrolysis process affected the condensation more than the aromatization degree of BC_MED_ vs. BC_LAB_.

### Stability in laboratory incubations

Laboratory incubations confirmed the high stability of BC_MED_, which was suggested by H/C ratio, BPCA and chemical oxidation methods. BC_MED_ mineralized by only 0.10% after 90 days, which is consistent with results of Luo and colleagues [38] who observed a 0.16% mineralization of 700°C *Miscanthus* biochars in an 87-day incubation. Lower temperature *Miscanthus* biochars have been reported to display higher mineralization rates, from 0.73% in 87 days for a 350°C biochar [38] to 1.1% in 200 days for a 575°C biochar [39]. Here, we could not estimate a precise MRTs based on our short-time laboratory incubation, but even the most conservative first-order kinetics model suggested it to be longer than 220 years (S1 Fig). Even if a laboratory MRT could be obtained it could not be extrapolated to field conditions, notably because incubation conditions are artificial and we used a standard soil type. Living roots can promote biochar mineralization [17] and soil type affects biochar mineralization rates [40]. What the incubations tell us is that BC_MED_ is highly stable and therefore worthy of field investigation. Incubations are also useful to compare the decomposition kinetics of different biochars [30]. Here we show that the stability of *Miscanthus* biochar produced in a medium-scale pyrolyzer actually exceeds that of biochar produced at a laboratory scale, which suggests that the large volume of feedstock in the pyrolyzer was not a limitation for obtaining a well carbonized product.

### Mineralization of BC in a two-year field trial

Mineralization rate of BC_MED_ in the field approximated 0.5% per growing season (Table 6), which implies that the annual rate is probably lower than 1% for the entire year under the cold-climate conditions prevailing in Norway. We acknowledge that the average 0.5% mineralization rate per growing season is only an estimate. However, we found no obvious source of bias on this estimate and therefore consider it fairly robust. Although our soil respiration fluxes were obtained with a simple manual chamber system, our results appear consistent with literature values. We measured on average a soil CO_2_ efflux of about 275 g CO_2_-C m^-2^ over 4 months in 2012, while the annual soil respiration from all croplands averages 544 g C m^-2^ yr^-1^ [41]. Our soil respiration data appear similar or higher to those compiled for field crops in Sweden, Canada and Russia [42].

For soil respiration alone, the absence of a significant difference between our biochar treatments and the control appears consistent with recent reports. For example, Schimmelpfennig and colleagues [43] report that throughout an 18-month monitoring period, a field having received *Miscanthus* biochar had lower cumulative CO_2_ emissions than biochar-free controls. In a recent meta-analysis, Sagrilo and colleagues [15] indicate that soil CO_2_ efflux from biochar treated soils are not significantly higher than from no-biochar controls when the ratio of biochar-C to SOC is lower than 2. Across application dose, these authors report no increase in soil CO_2_ efflux with biochar addition when the biochar is produced with a pyrolysis retention time > 30 minutes or at a temperature above 550°C, or when it has a surface area > 50 m^2^ g^-1^. In addition, none of the 8 field studies included in the review of Sagrilo and colleagues [15] displayed significant higher CO_2_ fluxes with biochar addition to soil. These findings suggest that biochar decomposition in the field is slow. However, actual quantification of the decomposition rate is crucial, as there is for example a large difference between a 1% and a 5% biochar decomposition rate, although both are likely to produce non-significant CO_2_ responses in the field, being possibly hidden by negative priming effects and root respiration responses. Therefore, isotopic tracing of C sources is needed to estimate the actual biochar mineralization rate in the field [16], as was conducted for one growing season in the present study.

Our biochar mineralization estimates computed from δ^13^C and soil respiration measurements are in the lower range of the limited set of studies having attempted a similar assessment. A mineralization rate of 9% was reported for maize biochar after 245 days [17]. However, biochar in the latter study had an atomic H/C ratio of 0.49, which is higher than our 0.18 value. In Australia, mineralization rates of *Eucalyptus* biochar ranged from 2% to 7% per year depending on soil type and climate [40]. This high mineralization rate might be due to the high H/C ratio of the *Eucalyptus* biochar, i.e. 0.63, which is higher than the H/C threshold of 0.6 for proposed for non-stable biochars [23]. Our results are similar to those of Major and colleagues [44], who reported a biochar mineralization rate of 2.2% over 2 years, i.e. about 1% per year, in tropical conditions, using a biochar made of mango tree wood with H/C atomic ratio of 0.26. Also, Maestrini and colleagues [45] reported an *in situ* annual mineralization rate of 0.5% for pinewood biochar in a temperate forest soil.

Estimating a MRT from the measured biochar mineralization rate in the field is the most crucial yet most uncertain step for assessing the C-storage potential of different biochar products in soil. Having measured a 2% mineralization for biochar over 12 months in an arenosol, Singh and colleagues [40] applied one-, two- and infinite-pool decomposition models and inferred that the corresponding MRT was comprised between 44 and 1079 years, which clearly exemplifies the large uncertainty associated with converting annual mineralization rates into MRT. Major and colleagues [44] observed a mineralization rate of 2.2% over two years, and extrapolated this value to a MRT of 3200 years using a two-pool model. This long MRT was a result of a three-fold decrease in biochar mineralization rate from year one to year two in their study. Our estimated mineralization rate for the 2012 season was slightly lower than that of Major and colleagues [44], i.e. 0.8 vs. 1.1% per year. However, we cannot apply a two-pool model to our results because we have no indication that such two pools actually existed in our case. Laboratory incubation (Fig 1) did not reveal any significant pool of mineralizable C for BC_MED_ at the beginning of the incubation. By contrast, the feedstock displayed a pronounced two-pool behavior, with 45% being mineralized in 90 days, which might explain why feedstock mineralization rates in the field in 2012 were fairly low. We used a one-pool model with constant mineralization rate of 0.8% per year, which yields a conservative MRT estimate for BC_MED_ of 125 years. Although this value barely exceeds the conventional 100-year threshold for permanent removal, large gains in terms of C storage in soil can still be achieved with a pyrolysis process transforming crop residues into biochar with 1% y^-1^ mineralization rate [46].

In conclusion, our biochar produced in a medium-scale pyrolyzer: 1) scored high on stability indices in the laboratory, 2) had similar to higher stability indices than a laboratory-produced biochar, and 3) mineralized at an estimated 0.8% per year under field conditions. The corresponding MRT for field conditions exceeds 100 years, but is only an extrapolation. Based on laboratory re-incubations, Spokas [47] argues that field-incorporated biochar might become intrinsically more susceptible to mineralization. Others have argued the opposite, that the real MRT might greatly exceed the projected MRT because biochar is not composed of one or two pools but of a continuum of increasingly recalcitrant fractions [40]. Ascertaining the long-term dynamics of this response calls for long-term monitoring of biochar field experiments having isotopic C tracing possibilities.

## Supporting information

S1 Fig Modelling with one-pool first-order kinetics models of the mineralization rate of BCMED in laboratory incubation. (PDF)

S2 Fig Example of Keeling plot obtained for the determination of the δ13C value of the soil CO2 in plots having received 8 t Miscanthus-C ha-1 (MS8) or 25 t biochar-C ha-1 (BC25). (PDF)

S1 Table Two-way ANOVA factorial analysis of biochar type × dose effects on cumulative mineralization after 90 days. (PDF)

S2 Table Two-way ANOVA factorial analysis of biochar type × dose effects on cumulative priming after 90 days. (PDF)

S3 Table Crop yields in 2011 and 2012 for control (C), 8 t biochar-C ha^-1^ (BC8), 25 t biochar-C ha^-1^ (BC25) and 8 t *Miscanthus*-C ha^-1^ (MS8). (PDF)

S4 Table Analysis of variance for average δ13C of soil CO2 in 2012 in the biochar plots (averaged of BC25 and BC8) vs. control plots. (PDF)

## Abbreviations

BPCA: benzene polycarboxylic acid
HTT: highest treatment temperature
MRT: mean residence time
SOM: soil organic matter
TOC: total organic carbon
