# Supplementary material for: Persistence in soil of Miscanthus biochar in laboratory and field conditions
Source: PLoS One. 2017 Sep 5;12(9):e0184383. doi: 10.1371/journal.pone.0184383 (PMC5584961; doi:10.1371/journal.pone.0184383)
Supplement: S1 Fig — (PDF) [file pone.0184383.s001.pdf]

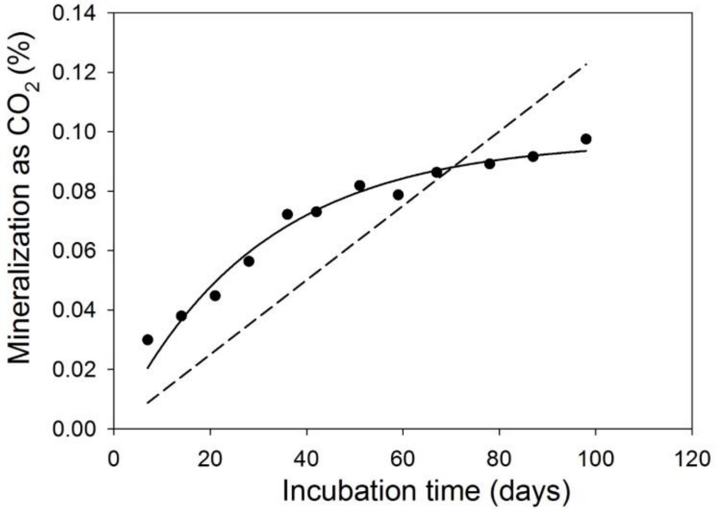

S1 Fig. Modelling with one-pool first-order kinetics models of the mineralization rate of BCMED in laboratory incubation. Dots are data. Solid line: mineralization % = 0.097% × (1- exp(-0.034 × days)). Dashed line: mineralization % = 100.00% × (1 - exp(-0.0000125 × Days))
