# Supplementary material for: Persistence in soil of Miscanthus biochar in laboratory and field conditions
Source: PLoS One. 2017 Sep 5;12(9):e0184383. doi: 10.1371/journal.pone.0184383 (PMC5584961; doi:10.1371/journal.pone.0184383)
Supplement: S2 Fig — (PDF) [file pone.0184383.s002.pdf]

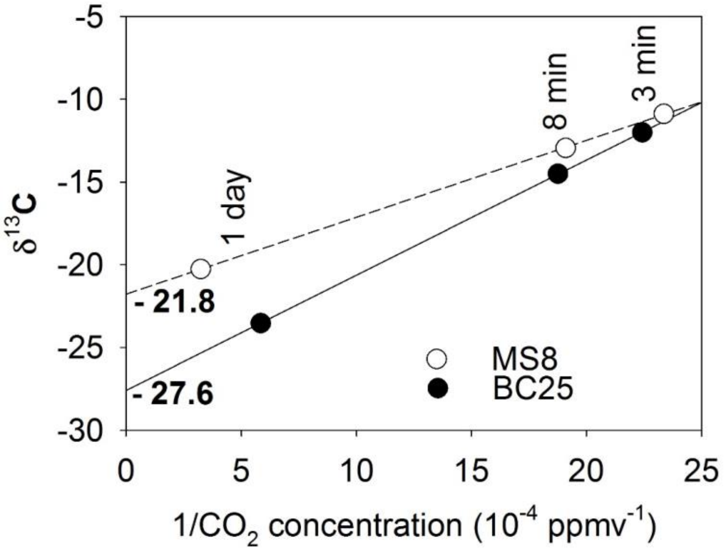

S2 Fig. Example of Keeling plot obtained for the determination of the $\delta^{13}C$ value of the soil CO_2 in plots having received 8 t *Miscanthus*-C ha^{-1} (MS8) or 25 t biochar-C ha^{-1} (BC25).
