# Supplementary material for: Persistence in soil of Miscanthus biochar in laboratory and field conditions
Source: PLoS One. 2017 Sep 5;12(9):e0184383. doi: 10.1371/journal.pone.0184383 (PMC5584961; doi:10.1371/journal.pone.0184383)
Supplement: S1 Table — (PDF) [file pone.0184383.s003.pdf]

S1 Table. Two-way ANOVA factorial analysis of biochar type × dose effects on cumulative mineralization after 90 days. Biochar type: BC_{MED} and BC_{LAB}; biochar dose: 0.23, 1.14 or 5.46 % by weight.

Source of Variation	*F*	*P*
Type	5.77	0.033
Dose	1.69	0.226
Type × Dose	1.34	0.291
