# Supplementary material for: Persistence in soil of Miscanthus biochar in laboratory and field conditions
Source: PLoS One. 2017 Sep 5;12(9):e0184383. doi: 10.1371/journal.pone.0184383 (PMC5584961; doi:10.1371/journal.pone.0184383)
Supplement: S2 Table — (PDF) [file pone.0184383.s004.pdf]

S2 Table. Two-way ANOVA factorial analysis of biochar type × dose effects on cumulative priming after 90 days. Amendment type: *Miscanthus* feedstock (MS), BC_{MED} and BC_{LAB}; amendment dose: high (5.5% BC_{MED} and BC_{LAB} and 0.58% MS) and low (1.1% BC_{MED} and BC_{LAB} and 0.12% MS).

Source of Variation	*F*	*P*
Type	190	<0.001
Dose	1321	<0.001
Type × Dose	80	<0.001
