# Supplementary material for: Persistence in soil of Miscanthus biochar in laboratory and field conditions
Source: PLoS One. 2017 Sep 5;12(9):e0184383. doi: 10.1371/journal.pone.0184383 (PMC5584961; doi:10.1371/journal.pone.0184383)
Supplement: S3 Table — (PDF) [file pone.0184383.s005.pdf]

S3 Table. Crop yields in 2011 and 2012 for control (C), 8 t biochar-C ha^{-1} (BC8), 25 t biochar-C ha^{-1} (BC25) and 8 t *Miscanthus*-C ha^{-1} (MS8). Averages and standard deviations for n = 4. DM = dry matter. No significant difference observed among treatments according to ANOVA analysis for 2011 (P = 0.37) and 2012 (P = 0.88).

Treatment	Oats 2011	Barley 2012
	t DM ha^{-1}	
C	5.33 (±0.73)	3.76 (±0.94)
BC8	5.13 (±0.18)	4.07 (±0.65)
BC25	5.64 (±0.55)	3.96 (±0.73)
MS8	5.24 (±0.49)	3.77 (±0.45)
