# Supplementary material for: Persistence in soil of Miscanthus biochar in laboratory and field conditions
Source: PLoS One. 2017 Sep 5;12(9):e0184383. doi: 10.1371/journal.pone.0184383 (PMC5584961; doi:10.1371/journal.pone.0184383)
Supplement: S4 Table — (PDF) [file pone.0184383.s006.pdf]

S4 Table. Analysis of variance for average $\delta^{13}C$ of soil CO_2 in 2012 in the biochar plots (averaged of BC25 and BC8) *vs*. control plots (n = 4).

Source of Variation	*F*	*P*
Biochar	8.23	0.064
Block	2.66	0.222
